# Doped Calcium Silicate Ceramics: A New Class of Candidates for Synthetic Bone Substitutes

**DOI:** 10.3390/ma10020153

**Published:** 2017-02-10

**Authors:** Young Jung No, Jiao Jiao Li, Hala Zreiqat

**Affiliations:** Biomaterials and Tissue Engineering Research Unit, School of AMME, University of Sydney, Sydney 2006, Australia; yono7153@uni.sydney.edu.au (Y.J.N.); jiaojiao.li@sydney.edu.au (J.J.L.)

**Keywords:** calcium silicate, bioactive ceramic, synthetic bone substitute

## Abstract

Doped calcium silicate ceramics (DCSCs) have recently gained immense interest as a new class of candidates for the treatment of bone defects. Although calcium phosphates and bioactive glasses have remained the mainstream of ceramic bone substitutes, their clinical use is limited by suboptimal mechanical properties. DCSCs are a class of calcium silicate ceramics which are developed through the ionic substitution of calcium ions, the incorporation of metal oxides into the base binary *x*CaO–*y*SiO_2_ system, or a combination of both. Due to their unique compositions and ability to release bioactive ions, DCSCs exhibit enhanced mechanical and biological properties. Such characteristics offer significant advantages over existing ceramic bone substitutes, and underline the future potential of adopting DCSCs for clinical use in bone reconstruction to produce improved outcomes. This review will discuss the effects of different dopant elements and oxides on the characteristics of DCSCs for applications in bone repair, including mechanical properties, degradation and ion release characteristics, radiopacity, and biological activity (in vitro and in vivo). Recent advances in the development of DCSCs for broader clinical applications will also be discussed, including DCSC composites, coated DCSC scaffolds and DCSC-coated metal implants.

## 1. Introduction

Over 2.2 million bone graft procedures are performed annually worldwide for the repair of bone defects arising from trauma or disease [1]. However, the successful reconstruction of large bone defects using conventional autograft and allograft transplantation has remained a clinical challenge. Autografts, although considered the current gold standard of graft materials, suffer from significant limitations including the requirement for second surgery, donor site morbidity, limited available bone volume for resection, and considerable graft resorption [2,3,4]. Allografts are restricted by the risk of disease transmission, reliance on donors [4,5], and reduced bioactivity of the graft due to the decellularisation procedures necessary to remove graft immunogenicity [5,6]. Therefore, an urgent need exists for the development of purely synthetic, readily available, and off-the-shelf bone substitutes with the same desirable characteristics as bone grafts but without their associated limitations, which will provide an alternative treatment option to produce improved outcomes of bone repair. A number of commercial bone substitutes are already used to replace bone grafts in orthopaedic procedures, but their widespread use remains limited as they do not satisfy many of the regenerative requirements of bone tissue and practical requirements relating to surgery and handling. An optimal bone substitute for achieving successful repair and regeneration in the clinical treatment of critical-sized bone defects should satisfy the following desirable criteria [7]:

- Ability to maintain in vivo mechanical stability at the defect site and withstand physiological loads.
- Radiopacity for easy implant monitoring using non-invasive methods such as X-ray and micro-computed tomography (µ-CT).
- Bioactivity to promote implant integration with host bone, as well as induce bone formation inside and surrounding the implant.
- Ability to be manufactured into macroporous scaffolds with high porosity and interconnectivity to promote bone ingrowth and vascularisation.
- Ability to degrade at a controlled rate that matches the rate of new bone formation.
- Ability to allow easy handling and sterilisation.

**Figure 1:**
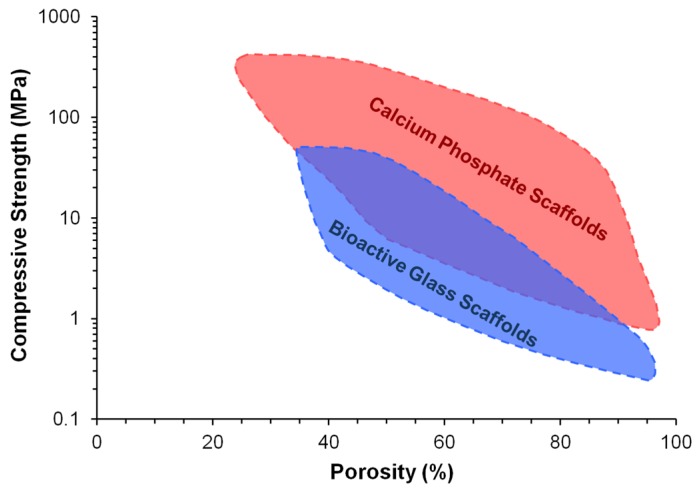
Dependence of compressive strength on porosity for bioactive ceramic scaffolds [16]. Reproduced by permission of the Royal Society of Chemistry, Copyright © 2014.

Bioactive and biodegradable ceramics have favourable properties for use as purely synthetic and off-the-shelf bone substitutes, as they resemble the mineral composition of bone and promote the formation of a direct bond with host bone without an intermediate fibrous tissue layer. A number of ceramic materials are already in clinical use to aid the repair of critical-sized bone defects, including hydroxyapatite, β-tricalcium phosphate (β-TCP), and biphasic calcium phosphate (BCP) in the calcium phosphate ceramic family, as well as Bioglass 45S5 and S53P4 in the bioactive glass family [8,9,10,11,12,13,14]. These materials are osteoconductive (allow the attachment and growth of bone-related cells on the surface), while bioactive glasses and some porous calcium phosphates are also osteoinductive (can actively induce new bone formation through biomolecular signalling and recruitment of osteoprogenitor cells) [15]. Nevertheless, despite their high bioactivity, current ceramic bone substitutes only have limited applications as macroporous blocks for the grafting of small bone defects or as particles for the filling of contained bone defects, and only at non-load bearing areas [16]. This is largely due to their poor mechanical strength and fracture toughness, which make them unsuitable for implantation in load-bearing regions [13,17]. The problem is exacerbated by the inverse relation between porosity and mechanical properties of ceramic materials (Figure 1). Although high porosity and interconnectivity are desirable properties for bone substitutes to produce enhanced outcomes of bone regeneration, clinically used ceramic materials often have little or no porosity in order to retain sufficient mechanical properties for implantation [16]. Further drawbacks associated with current ceramic bone substitutes include suboptimal radiopacity and degradation kinetics. Other than these common problems, calcium phosphate ceramics and bioactive glasses also each have their own disadvantages which have restricted their widespread clinical application. For example, unlike bioactive glasses, calcium phosphate ceramics generally do not possess intrinsic osteoinductivity and must rely on added surface porosities and concavities to capture circulating bone-forming cells and growth factors [18]. On the other hand, unlike calcium phosphate ceramics, bioactive glasses cannot be easily processed into porous scaffolds without losing their bioactivity due to crystallisation at high sintering temperatures [19].

Due to the problems encountered with clinically available ceramic bone substitutes composed of calcium phosphates and bioactive glasses, calcium silicate ceramics have gained intense research interest since the mid-2000s to provide a potential alternative. Pioneering work in this area began with studying the properties of pseudowollastonite (α-CaSiO_3_) or α-calcium silicate (α-CS), which was shown to be bioactive due to the ability to form a surface apatite layer when immersed in simulated body fluid (SBF) [20,21]. An interesting and useful property of α-CS is the ability to be fabricated into different bulk shapes and structures through high temperature sintering without compromising the bioactivity of the ceramic [22]. The potential of using α-CS for bone regeneration has been confirmed by a number of studies, which showed ability of the material to achieve direct bonding with native bone resulting in favourable in vitro and in vivo regeneration outcomes [21,23]. However, two major limitations of α-CS have prevented its development as a bone substitute for clinical use. Firstly, it has a high dissolution rate which generates a highly alkaline environment, resulting in alkaline-induced toxicity [24,25] and excessive release of Ca and Si ions to levels inhibitory to cell proliferation [26]. Secondly, the mechanical strength of α-CS as dense monoliths and porous scaffolds is very low compared to the ranges of values required for the regeneration of cortical and cancellous bone, which reduces the ability of the material to maintain mechanical stability under physiological loads [27,28,29]. Due to these limitations, researchers have been exploring strategies to optimise the degradation rate and improve the mechanical properties of calcium silicate ceramics. A highly successful approach is to incorporate various metal ions and/or metal oxides into the base α-CS crystal structure to produce doped calcium silicate ceramics (DCSCs). This review will compare the important properties of different DCSCs, including mechanical properties, degradation and ion release characteristics, and radiopacity, as well as discuss the available in vitro and in vivo evidence relating to the use of DCSCs for bone regeneration. Recent advances in the development of DCSCs for broader clinical applications will also be discussed, including DCSC composites, coated DCSC scaffolds and DCSC-coated metal implants. DCSCs included into this review have been selected based on the following criteria:

- Constitutes a crystalline material (hence excluding silicate-based bioactive glasses and glass-ceramics).
- Constitutes a monophasic material with a single identifiable crystalline phase.
- Contains a dopant which is (1) an element incorporated for ionic substitution of calcium; (2) a metal oxide incorporated into the *x*CaO–*y*SiO_2_ structure; or (3) a combination of both strategies.
- Has been tested for biocompatibility or bioactivity through at least one in vitro or in vivo experiment.

## 2. Synthesis of DCSCs

The stoichiometric formula, reported fabrication method and heat treatment for a range of monophasic DCSCs are presented in Table 1. These DCSCs have been produced by (1) ionic substitution of Ca with divalent ions such as Sr [30,31] and Cu [32]; (2) incorporation of metal oxides into the *x*CaO–*y*SiO_2_ structure [33,34,35,36,37,38,39,40]; or (3) a combination of both strategies [25,41,42]. The oxides used to dope calcium silicate typically contain a divalent (Sr, Zn, Mg, Cu, Co) or quadrivalent (Ti, Zr) metal ion, with the exception of aluminium oxide which forms gehlenite after doping [34]. The rationale behind using these particular metal oxides for doping is that they contain the same metallic elements as the trace elements found in bone, such as magnesium (Mg) [43], zinc (Zn) [44] and strontium (Sr) [44], which are known to have beneficial effects in promoting bone formation [45]. The use of other metal oxides containing titanium (Ti), zirconium (Zr) and aluminium (Al) for doping is based on the historical use of titanium alloys [46], zirconia [47] and alumina [48] as implantable orthopaedic materials. Titanium oxides in particular have been shown to enhance the bioactivity of hydroxyapatite [49] and bioactive glasses [50]. 

Chemical precipitation, sol-gel, and solid-state sintering are the three commonly used methods for preparing DCSC precursors prior to calcination. Although no studies have directly compared these methods for the synthesis of a particular DCSC, it has been noted that sol-gel has a relatively low powder yield, and that solid-state sintering requires the amount of volatile compounds to be precisely determined in the starting material [51]. Calcination is performed to induce reaction among the precursors to form the desired ceramic phase or phases, as well as to remove all organic residues from precursor fabrication. For DCSCs in particular, optimisation of the temperature treatment profile used for calcination is of primary importance for obtaining pure monophasic ceramics. A minimum calcination temperature exists that allows the desired ceramic phase to be fully obtained, below which incomplete phase transformation leads to a significant portion of undesirable impurities, often in the form of un-reacted or partially reacted precursors. For example, calcination of CaO–SiO_2_ at temperatures below 1200 °C results in the formation of β-CS rather than α-CS [31]. For hardystonite, lowering the calcination temperature to 1100 °C leads to the undesirable formation of an intermediate willemite phase (2ZnO-SiO_2_) [39]. For cuprorivaite, calcination temperatures outside the optimal 1000 °C lead to the formation of SiO_2_ and CuO [35], while Ca_2_SiO_4_ impurities appear for Co-akermanite below the optimal calcination temperature [42].

While an optimal calcination temperature is required to produce ceramic powders with the desired phase(s), the fabrication of ceramic materials, including DCSCs, into specific morphologies for end-use applications also requires sintering at an optimal temperature. At the optimal sintering temperature which is unique for different ceramics, ceramic powders which have been pressed or manipulated to form a specific shape can react with adjacent particles to form a defined physical structure. Below the optimal sintering temperature, inadequate densification of the ceramic structure leads to significant reduction in mechanical properties of the resulting construct. Above the optimal sintering temperature, the ceramic can have reduced mechanical strength due to increased grain size [34], or melt and therefore fail to form the predefined structure.

## 3. Mechanical Properties of Solid and Porous DCSCs

A key property of materials with intended application as synthetic bone substitutes is the ability to resist fracture when subjected to physiological loads. The brittle nature of ceramic materials is a primary hurdle restricting their widespread clinical use in bone reconstruction. Catastrophic failure in the load-bearing environment of a critical-sized defect almost invariably results in defect instability and disruption of the bone healing process. The reported mechanical properties (Young’s modulus, mechanical strength, and fracture toughness) for a range of DCSCs (including dense ceramic monoliths with porosity <20% and macroporous scaffolds with porosity >50%) are presented in Table 2. The values are compared with clinically used ceramic bone substitutes including Bioglass 45S5, hydroxyapatite, β-tricalcium phosphate (β-TCP), and biphasic calcium phosphate (BCP), as well as cortical and cancellous bone. The majority of DCSCs displayed significant improvements in mechanical properties compared to the clinically used ceramic bone substitutes, particularly for mechanical strength in bending and fracture toughness. Out of the DCSCs with reported mechanical properties, gehlenite and diopside exhibited the highest fracture toughness for dense monoliths, at 2.7 MPa·m^1/2^ [34] and 3.5 MPa·m^1/2^ [52] respectively, which greatly exceeded those of clinically used materials and reached the lower end of the reported range for cortical bone [7,13,17]. The other DCSCs showed bending strength in the range of 136–176 MPa and fracture toughness in the range of 1.2–1.8 MPa·m^1/2^, which were higher than the typical values observed for clinically used calcium phosphates and bioactive glasses.

The mechanical strength of dense DCSC disks is primarily determined by their sintering kinetics and densification profile, both of which are affected by the stoichiometric formula of the ceramic, the presence of dopant oxides, and the resulting atomic or ionic interactions. Similar relations have been observed for β-TCP scaffolds [53,54,55,56] and other ceramics [57,58,59] containing various oxide dopants. As shown in Table 2, all dense DCSCs (except hardystonite) had lower porosity compared to α-CS (15.5%) and β-CS (18.6%), indicating enhanced densification in DCSCs which is a key contributor to their improved mechanical strength. Variations in strength among different types of DCSCs could be attributed to other factors, such as differences in the crystal structure and ionic interactions between the dopant ions and oxides with the base *x*CaO–*y*SiO_2_ system.

The mechanical strength of macroporous DCSC scaffolds is largely affected by the porosity of the scaffold. DCSC scaffolds exhibit the same trend as that for other ceramic materials, where their mechanical strength is inversely proportional to porosity [16]. This creates a significant challenge for the design and fabrication of ceramic scaffolds for use in bone reconstruction. Sufficient mechanical strength is obviously a prerequisite for maintaining defect stability and providing adequate mechanical support for bone regeneration after scaffold implantation. However, scaffold porosity is also an important parameter due to its role in facilitating vascularisation and bone ingrowth to achieve bridging and reconstruction of the defect. Furthermore, the presence of macroporosity can reduce the high stiffness of the ceramic compared to its bulk form, thereby minimising the effects of stress shielding due to stiffness mismatch between the scaffold implant and bone. The current consensus in the essential features of pore geometry to achieve optimal bone regeneration are (1) fully interconnected pores; (2) pore sizes which are at least 100 µm in diameter; and (3) porosity that is as high as practically possible [60,61]. DCSCs face the same dilemma as that for other ceramic materials used for bone regeneration, in that the fabrication of scaffolds with both high strength and high porosity remains a prominent challenge. Compared to other DCSCs, hardystonite and Sr-hardystonite achieved the highest compressive strength for macroporous scaffolds (~2 MPa) at porosities exceeding 75% [25], but are still insufficient for matching the mechanical properties of cancellous bone.

### Influence of Fabrication Method on the Mechanical Properties of DCSC Scaffolds

Different fabrication methods can have a significant influence on the geometry and mechanical properties of ceramic scaffolds prepared from the same base material. Due to the requirement for ceramic sintering to form constructs with a defined three-dimensional architecture, popular methods for the fabrication of ceramic scaffolds are currently limited to the polymer sponge sacrificial template (PSST) technique and 3D printing by direct ink writing (DIW) or selective laser sintering (SLS). Other methods such as freeze-casting [62] and porogen leaching [30] have also been attempted for the fabrication of DCSC scaffolds, but may result in structures with limited pore interconnectivity. 

**Figure 2:**
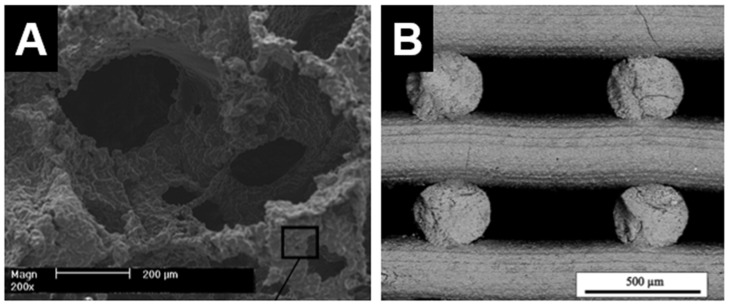
Internal structure of hardystonite scaffolds produced by (**A**) polymer sponge sacrificial template method [25]; and (**B**) 3D printing (direct ink writing) [65]. (**A**) Adapted by permission of Elsevier, Copyright © 2010; (**B**) adapted by permission of John Wiley and Sons, Copyright © 2016.

The PSST method has been commonly used for fabricating ceramics materials, including DCSCs, into macroporous scaffolds. The advantage of this method is the relative ease of fabrication and ability to generate highly porous and interconnected structures with geometry similar to that of cancellous bone [16]. A sacrificial polymer (often polyurethane) foam is coated with ceramic slurry composed of ceramic powder and a binder solution, which is then subjected to a heat treatment to burn off all organic components prior to ceramic sintering. The result is a ceramic scaffold that replicates the original structure of the sacrificial foam (Figure 2A) [25]. However, this method does not allow precise control over pore geometry, and the manual nature of the process limits the potential for automation and scale-up which are necessary for clinical translation. 

Recent technological advances have generated intense interest in 3D printing methods, such as DIW and SLS, for ceramic scaffold fabrication. These methods have gained increasing popularity due to affordability of the necessary equipment and ability for set-up in a laboratory environment. Their main advantage compared to PSST is the ability to allow customised design of scaffold architecture and pore geometry, as well as precise control over the fabrication process to produce the desired structures. These features are useful not only for producing customised scaffolds for end-use applications, but also for facilitating basic science research into scaffold characteristics which are important in modulating the bone regeneration process, such as pore geometry, gradient structures, and permeability. However, a primary disadvantage of 3D printing methods compared to PSST is the current inability to produce scaffolds with very high porosities exceeding 80%. For example, akermanite scaffolds produced by 3D printing were able to attain very high compressive strengths, but exhibited relatively low porosities of 50%–60% [63,64]. Hardystonite scaffolds with 75% porosity could be produced by DIW, but compressive strength was slightly lower than those with similar porosity produced by PSST (Figure 2B) [65]. The low porosity of 3D-printed ceramic scaffolds is primarily due to current limitations in the fabrication process, where the inability to adjust strut thickness below a certain threshold results in relatively thick struts and therefore reduced porosity. For example, the laser beam diameter for akemanite scaffolds printed by SLS was 1.0 mm, producing thick struts and hence low porosities of <60% [63]. Akemanite scaffolds printed by DIW also showed similar porosities [64]. In this method, the stability of the struts prior to sintering is directly dependent on the viscosity of the ink, necessitating a high strut thickness and also special formulation of the ink such that it is semi-solid upon extrusion. Extensive optimisation is therefore required for producing 3D-printed DCSC scaffolds with an optimal balance of strength and porosity, and some work has already been performed in this area using other types of ceramic materials [66,67].

## 4. Degradation and Ion Release Characteristics of DCSCs

An important property of bioactive ceramics enabling their use as synthetic bone substitutes is the ability to undergo controlled biodegradation, and release ions into the environment which contribute to inducing osteogenesis. The weight loss, pH, apatite formation and ion release characteristics in aqueous media of a range of DCSCs as observed during in vitro studies are presented in Table 3. The common time point chosen for comparison is 7 days, for which the majority of reviewed studies have reported degradation and ion release data. Care must be taken when interpreting and comparing the results of in vitro degradation studies, which are greatly affected by the experimental protocol and type of aqueous medium chosen. For most bioactive ceramics including DCSCs, the ion release kinetics is often non-linear and depends on the concentration gradient between the ceramic surface and composition of the surrounding medium. In the majority of cases, an initial burst release of ions is observed during the first few days, followed by a plateau if the experimental protocol does not involve replenishing the surrounding medium. The same ion release profile is unlikely to be obtained in vivo, due to the constant flow of interstitial fluid in a highly homeostatic environment. Nevertheless, the investigation of in vitro degradation and ion release are important for initial assessment of a bioactive ceramic prior to conducting in vivo studies.

**Figure 3:**
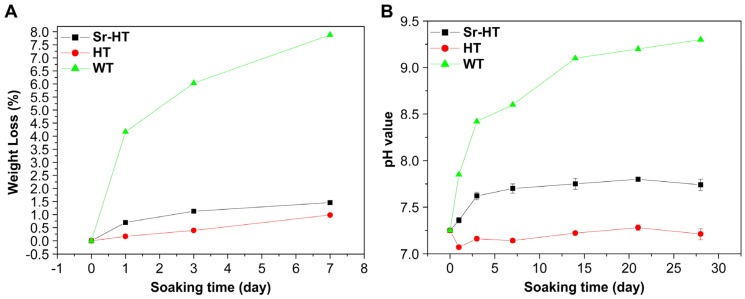
Compared to calcium silicate (WT) scaffolds, Sr-hardystonite (Sr-HT) and hardystonite (HT) scaffolds immersed in simulated body fluid showed (**A**) reduced weight loss and (**B**) smaller pH changes [25]. These trends were representative of those exhibited by other types of DCSCs in degradation experiments. Reproduced by permission of Elsevier, Copyright © 2010.

A primary requirement for DCSCs with intended application in bone reconstruction is the ability to undergo controlled biodegradation in physiological fluids, and be gradually replaced by new bone tissue. In general, the results across a range of experimental studies all indicated reduced weight loss, ion release and alkalinity for different types of DCSCs compared to calcium silicate when tested under similar conditions (Figure 3). This is highly favourable for the application of DCSCs as synthetic bone substitutes, as rapid dissolution and the tendency to create a highly alkaline environment are the major drawbacks limiting the use of calcium silicates. For example, α-CS scaffolds have been shown to undergo degradation up to 7–11 wt % in 7 days and raise the surrounding pH to 8.1–8.6 [29]. A number of DCSCs were directly compared with α-CS controls in the same experiment, all of which exhibited lower weight loss, pH change and ion release, including Sr-α-CaSiO_3_ [31], hardystonite [25], Sr-hardystonite [25], sphene [40], and baghdadite [33]. Interestingly, a number of DCSCs including diopside [36,70], sphene [40], hardystonite [25] and gehlenite [34] exhibited almost no weight loss after 7 days (<1 wt %), with surrounding pH in the range of 7.2–7.7. These DCSCs might be useful in bone reconstruction at defect sites which require mechanical stability to be maintained by the implant for an extended period of time. For DCSCs doped with the Sr ion, their degradation and ion release characteristics were largely affected by the mol% of Sr ion substitution as in the case of Sr-α-CS [31], or the presence of additional dopant oxides as in the case of Sr-hardystonite [25]. Of all reviewed DCSCs, only baghdadite exhibited weight loss (~9 wt % after 7 days) similar to α-CS, but induced only slight increases in the surrounding pH to 7.5–8.0 [71]. Baghdadite ceramics might therefore be used in applications requiring rapid dissolution and high bioactivity of the scaffold implant to induce accelerated bone formation at the defect site, without raising the pH to toxic levels. For future investigations into the in vitro degradation behaviour of DCSCs, it might be of interest to conduct tests under acidic conditions such as using citric acid buffer solution, which has already been performed with gehlenite [34]. This will imitate the local acidification created by osteoclasts and macrophages during the bone remodelling process, which is an additional contributor to ceramic dissolution due to its role in facilitating cell-mediated resorption [75,76].

Comparing the ion release rates of DCSCs doped with various metal ions or oxides revealed several interesting observations. DCSCs doped with Zn, Ti and Zr showed release rates of these transitional metal ions which were orders of magnitude lower than the release rates of Mg, Sr and Al from other DCSCs under similar experimental conditions. At the same time, some DCSCs showed release rates of dopant ions which were independent of Ca and Si release from the same ceramic. For instance, baghdadite showed very low levels of Zr release, but one of the highest release rates for Ca and Si compared to other DCSCs [33], while gehlenite showed appreciable release of Al ions but one of the lowest release rates for Ca and Si [34]. Sphene and hardystonite, doped respectively with Ti and Zn, showed the lowest overall ion release rates [25,40]. The dissolution rate of DCSCs in an aqueous environment is generally a function of the dopant ion valency [77] and metal-oxide bonding strength [78], although the exact roles of dopant ions and oxides in modulating the in vitro degradation behaviour of DCSCs requires further investigation.

Almost all DCSCs have the ability to form a surface apatite or apatite-like layer after immersion in simulated body fluid (SBF). A large volume of existing literature has considered this ability of a material to form a surface apatite layer in SBF as an indicator of ‘in vitro bioactivity’, due to evidence that native bone tissue can integrate with the apatite layer in vivo and hence form a strong chemical bond with the implanted material [79]. This mechanism of bioactivity is best exhibited by most bioactive glasses [10]. Although in vitro apatite formation is a potent indicator of material bioactivity, scientific advances have resulted in the widespread use of cell culture techniques as a more accurate method for evaluating in vitro bioactivity. For this reason, in vitro apatite formation is now more commonly used as a tool for understanding the mechanism, rather than being the sole indicator, of material bioactivity [80]. Some DCSCs, such as hardystonite [81], sphene [40], and gehlenite [34], are clearly bioactive as shown by in vitro cell experiments, but do not induce surface apatite formation. The development of surface apatite relies on the formation of a silica gel, which acts as a platform for the deposition of extracellular calcium ions and formation of nucleation sites for apatite mineralisation [82,83]. Interestingly, hardystonite, sphene and gehlenite showed the lowest release of Si ions after 7 days (5–6 ppm) compared to calcium silicate and other DCSCs [34,40,81], which might explain their lack of surface apatite layer formation. In these ceramics, bioactive ion release and microstructural characteristics are likely to be the primary mechanisms of bioactivity.

## 5. Radiopacity of DCSCs

An often overlooked consequence of incorporating metal oxides into the *x*CaO–*y*SiO_2_ crystal structure to produce DCSCs is the change in X-ray mass attenuation coefficient (µ/ρ, XMAC) of the ceramic. Few studies in the literature have explored and compared the radiopacity of DCSCs, although one study showed a linear improvement in radiopacity by incorporating increasing amounts of baghdadite into a polymer matrix [86]. The XMAC of a compound depends on its elemental composition, as heavier elements tend to have higher XMAC values. The clinical relevance of this is that a higher XMAC indicates increased radiopacity and therefore visibility of the material using non-invasive methods such as X-ray and µ-CT. The exact XMAC value of a compound depends on the energy of the X-ray used, and can be calculated by summing the products of the elemental XMAC and weight fraction of the elements within the stoichiometric formula [87]:

(1) $${\mathrm{XMAC}}_{\mathrm{compound}}=\underset{i}{\sum }w_{i}{\mathrm{XMAC}}_{i}$$

In the above equation, w*_i_* and XMAC*_i_* are the weight fraction and XMAC of the *i*th constituent, respectively. This equation allows calculation and comparison of the XMAC value of different materials at a given X-ray energy, and the relative ranking of materials based on their XMAC tends to remain identical at other X-ray energies. Using elemental XMAC values [87], the calculated XMAC at 20 keV energy for a range of DCSCs are presented in Table 4, along with the XMAC for Bioglass 45S5, hydroxyapatite, tricalcium phosphate, calcium silicate, and cortical bone [87] which have been included for comparison. Although the XMAC values in Table 4 are based on dense materials, the corresponding XMAC for porous structures composed of the same materials can be easily calculated. Under the reasonable assumption that the XMAC of air is negligible (0.778 at 20 keV [87]) compared to the ceramic, a conservative estimate of the XMAC for a porous scaffold of a particular material can be obtained by simply multiplying the XMAC of the dense material by (1-porosity).

In a clinical setting, radiopacity of an implant material with intended use as a synthetic bone substitute is a highly important property that is often overlooked during material design and characterisation. For scaffold implants used in bone reconstruction, sufficient radiopacity to provide contrast from the bone tissue will allow the clinician to easily examine implant interactions with the surrounding tissue, as well as to monitor implant resorption over time. Currently, implant materials which are not radiopaque but require X-ray visibility for clinical monitoring must rely on doping with bioinert particles which have a high XMAC, such as barium sulfate, tantalum oxide, and zirconia. This compensatory method has been applied in orthopaedics to polymer-based implants such as poly(methyl methacrylate) (PMMA) and calcium phosphate bone cements [88,89], and in dentistry to gutta percha [90]. From Table 4, it is evident that the majority of DCSCs have a XMAC that is significantly higher than cortical bone, which is an added advantage for their clinical application compared to existing bone substitute materials with XMAC values that are relatively close to bone, such as Bioglass 45S5, hydroxyapatite and tricalcium phosphate.

## 6. In Vitro Cell Interactions with DCSCs

The in vitro interactions of different DCSCs have been investigated using a range of cell types and material morphologies (powder extracts, dense disks and porous scaffolds), as shown in Table 5. The majority of studies reported enhanced cell proliferation and expression of genes related to osteogenesis and angiogenesis in the presence of DCSCs, with variations among different ceramics determined by the types of ions released and their concentrations or release rates. A range of bioactive ions are released from DCSCs that have important roles in promoting bone formation (Ca, Si, Sr, Mg, Zn) and angiogenesis (Mg, Cu, Co), both of which are vital processes in the successful reconstruction of vascularised bone tissue [45,91,92,93]. The favourable in vitro interactions of DCSCs with cell types relevant for bone regeneration support the development of these ceramics as synthetic bone substitutes. 

The in vitro cell interactions with DCSCs, as indicated by attachment, proliferation, gene expression and enzyme activity, are influenced by the amount of ceramic present but do not exhibit a linear dose-response relationship. Optimal cell interactions are often observed within a specific range of ion concentrations for a particular ceramic, which can be controlled for experiments performed using ceramic powder extracts by serial dilution of the extract solution. The highest extract concentration is usually 200 mg/mL, at which most DCSCs do not exhibit inhibitory or cytotoxic effects on cells. As shown in Table 5, cell activity is generally enhanced on dense disks and porous scaffolds of DCSCs, as well as ceramic powder extracts within a specific range of concentrations, compared to calcium silicate controls. Only cuprorivaite [35] and Co-akermanite [42] showed some cytotoxic effects due to Cu and Co release, respectively, to certain concentrations, while high extract concentrations of akermanite caused slight inhibition of proliferation in human adipose-derived stem cells [94]. For dense disks and porous scaffolds of DCSCs, the mechanism underlying enhanced cell activity is likely the combination of bioactive ion release and surface characteristics of the sample, including surface chemistry, topography and microstructure, with DCSC scaffolds providing an additional dimension of macroporosity.

**Figure 4:**
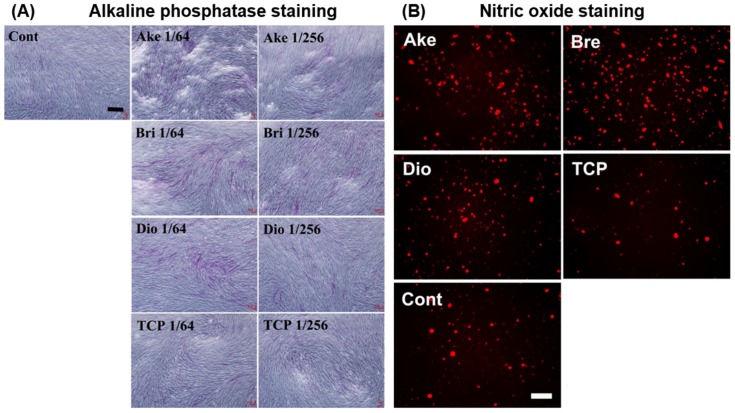
Ceramic powder extracts of akermanite, bredigite and diopside showed enhanced ability to promote (**A**) osteogenesis in human bone marrow mesenchymal stem cells, as demonstrated by staining for alkaline phosphatase; and (**B**) angiogenesis in human aortic endothelial cells, as demonstrated by staining for nitric oxide, compared to β-tricalcium phosphate and ceramic-free controls [101]. Cont: ceramic-free control, Ake: akermanite, Bri: bredigite, TCP: β-tricalcium phosphate. Adapted by permission of Elsevier, Copyright © 2013.

The majority of in vitro studies have implicated bioactive ion release as the primary mechanism leading to enhanced cell interactions with DCSCs. Sr ions released from Sr-α-CaSiO_3_, Sr-hardystonite, and Sr-baghdadite were found to enhance osteoblast proliferation and osteogenic gene expression compared to control ceramics without strontium [25,31,41]. Mg ions released from akermanite, diopside and bredigite promoted osteogenic gene expression in a range of cell types including human periodontal ligament cells [85,95,96], human induced pluripotent stem cells [97], and several types of adult stem cells capable of developing the osteoblast phenotype (Figure 4A) [69,94,98,99,100,101]. In addition, Mg ion release was found to enhance in vitro angiogenesis by human aortic endothelial cells (Figure 4B) [101]. The release of Zn ions from hardystonite led to enhanced attachment, proliferation and osteogenic gene expression of human osteoblasts [25,28,81] and bone marrow-derived mesenchymal stem cells [95,102]. Co [42] and Cu [32,35] ions released from several DCSCs were shown to have positive effects in inducing endothelial cell proliferation, angiogenic gene expression and in vitro angiogenesis at optimised concentrations. 

**Figure 5:**
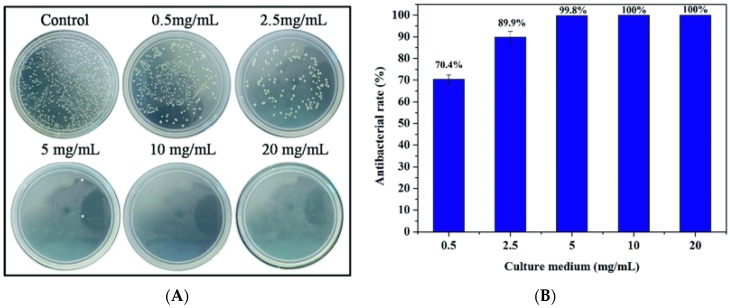
Cuprorivaite powder extracts showed significant ability to (**A**) inhibit the growth of *Escherichia coli* at certain concentrations, and (**B**) their antibacterial activity also exhibited a dose-dependent relation [35]. Adapted by permission of the Royal Society of Chemistry, Copyright © 2016.

An interesting and very useful property of some DCSCs revealed through in vitro experiments is antibacterial activity. For example, hardystonite extracts demonstrated ability to inhibit the proliferation of *Enterococcus faecalis* to a similar extent as calcium hydroxide [95], and cuprorivaite also showed antibacterial effects against *Escherichia coli* (Figure 5) [35]. The antibacterial activity of these DCSCs is the result of certain ions released into the environment, such as Zn and Cu. Similar properties are likely to be present in other DCSCs which can release ions known to have antibacterial effects, and represent an added advantage for their clinical use compared to currently available ceramic bone substitutes.

A number of in vitro studies have elucidated the mechanisms of enhanced osteogenesis and/or angiogenesis due to bioactive ion release from DCSCs. The extracellular signal-regulated kinase (ERK), Wnt/β-catenin, and bone morphogenetic protein (BMP)-2 signalling pathways are thought to be important mediators of cell interactions with DCSCs, although these have only been demonstrated with akermanite [94,100] and baghdadite [103]. Akermanite has additionally been shown to activate the p38, AKT and STAT3 signalling pathways with evidence of crosstalk among these pathways, which have downstream effects in promoting osteogenesis and angiogenesis [100]. Aside from studies performed on specific DCSCs, a large body of evidence exists in the literature on the positive roles of certain bioactive ions in inducing processes related to bone regeneration that could be used to explain the biological effects of DCSCs. For example, Ca and Sr ions can activate calcium sensing receptors and their downstream signalling pathways in osteoblasts [104,105], Si ions have an important role in the Wnt and Sonic Hedgehog (SHH) signalling pathways [106], and Mg ions can upregulate the Ras-MAP kinase signalling pathway [107].

Although in vitro cell experiments have provided valuable information on the nature and mechanisms of cell interactions with DCSCs, care should be taken when interpreting the results or using the results as evidence to predict the clinical performance of the material. Many in vitro experiments involving DCSCs tested cell responses to ceramic powder extracts, and evaluated these responses at certain concentrations of released ions. However, such controlled in vitro conditions hardly replicate the highly dynamic in vivo environment, where the ceramic would be subjected to constant fluid flow and a complex milieu of cells, biochemical factors and mechanical stresses which would all affect its degradation and ion release. As an example, akermanite and bredigite were shown to have enhanced in vitro angiogenic properties compared to diopside, despite all three ceramics possessing the same CaO–MgO–SiO_2_ base structure, which was thought to be the result of different concentrations of ions released from the ceramic extracts [101]. In another study, in vitro angiogenesis was shown to be enhanced when Cu ions existed concurrently with Ca and Si ions in the culture medium, but not when only Cu ions were present [35]. The results of these studies are useful for understanding the nature and mechanisms of cell responses to DCSCs, but have been obtained under controlled in vitro conditions which are very different from the actual in vivo conditions where ceramic dissolution will occur in clinical applications. Furthermore, the outcomes of in vitro experiments can be affected to a large extent by the cell type used, as different cell types can respond differently to various ions and concentrations of these ions. For example, human umbilical vein endothelial cells (HUVECs) could proliferate at higher Co-akermanite extract concentrations compared to MC3T3-E1 cells [42], human periodontal ligament cells responded better to diopside than hardystonite [95], akermanite inhibited rat bone marrow macrophage osteoclastogenesis but supported the proliferation of bone marrow-derived stem cells [100], and gehlenite supported both the proliferation of osteoblasts and maturation of osteoclasts [34]. In vivo studies are therefore necessary to enable more accurate prediction of the therapeutic efficacy of DCSCs in clinical applications.

## 7. In Vivo Performance of DCSCs

**Figure 6:**
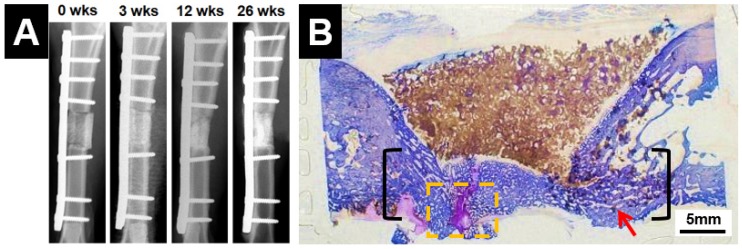
Baghdadite scaffolds achieved effective repair of a critical-sized segmental defect in the sheep tibia, with (**A**) radiographic evidence of clinical union at the bone-scaffold interface; and (**B**) histological evidence of significant and almost complete bridging of the defect, as well as bone infiltration and remodelling within the scaffolds [110]. Adapted by permission of IOP Publishing, Copyright © 2016.

The in vivo performance of several DCSCs (in the form of dense specimens or porous scaffolds) has been investigated, as shown in Table 6. In general, all studies demonstrated improved bone regeneration outcomes using DCSC implants compared to calcium silicate or calcium phosphate controls. The DCSC implants were well tolerated in both small and large animal models, with no evidence of inflammatory reactions or the formation of surrounding fibrous tissue. In particular, porous scaffolds of hardystonite [25], Sr-hardystonite [25] and baghdadite (Figure 6) [71,110] achieved complete or almost complete bridging of critical-sized defects by inducing the rapid growth of new bone from the defect borders towards the centre. In addition, these scaffold implants encouraged new bone growth into the macropores of the scaffold, thereby facilitating improved integration and interactions between the scaffold and host bone. This was in contrast to the calcium phosphate controls, for which bone growth was limited to the outside of the scaffold with minimal penetration of the scaffold pores. Sr-β-CS [30] and akermanite [100] scaffolds also demonstrated increased new bone volume and trabecular bone thickness, as well as enhanced in vivo degradation, compared to calcium silicate and calcium phosphate controls. 

The available in vivo evidence on the performance of DCSC implants in a range of orthotopic animal models suggests that DCSCs may achieve improved reconstructive outcomes in orthopaedic applications compared to current bone substitutes composed of calcium phosphates or bioactive glasses, which is supported by their favourable mechanical properties, degradation characteristics and ability to enhance cell interactions as discussed in previous sections. Nevertheless, a limited number of in vivo studies have been performed on DCSCs to date, which mostly involve small animal models (rats and rabbits, with only one study performed in sheep [110]), relatively short implantation periods, and mostly macroscopic or structural evaluations of bone regeneration outcomes (by gross examination, radiography, µ-CT, and histology). Building on the available in vivo evidence, future preclinical studies evaluating the efficacy of DCSC implants to predict their clinical performance can provide more compelling information by (1) using large and clinically relevant animal models, and creating defects which resemble those commonly encountered in clinical situations; (2) conducting the study over longer time periods to evaluate the long-term outcomes of regeneration and in vivo degradation; and (3) performing functional and biochemical evaluations (such as biomechanical testing, gene and protein analyses) in addition to macroscopic and structural evaluations to enable full assessment of bone reconstruction outcomes. The results of these studies will propel the development of DCSCs as synthetic bone substitutes with improved properties for the clinical treatment of challenging bone defects.

## 8. Development of DCSCs for Broader Clinical Applications

Due to the unique combination of properties exhibited by DCSCs and their ability to encourage osteogenesis, recent research has focused on diversifying the potential applications of DCSCs by incorporating them into new material systems. DCSC-inorganic composites have been developed to create constructs with enhanced mechanical properties. Polymer-DCSC composites have been fabricated by using DCSCs for reinforcement inside a polymer matrix. Porous DCSC scaffolds have been coated with a thin polymer-based layer to produce improved strength and toughness. Finally, the potential of applying DCSCs as coatings on metal implants to enhance osseointegration has been explored using titanium and magnesium alloys.

### 8.1. DCSC-Inorganic Composites

**Figure 7:**
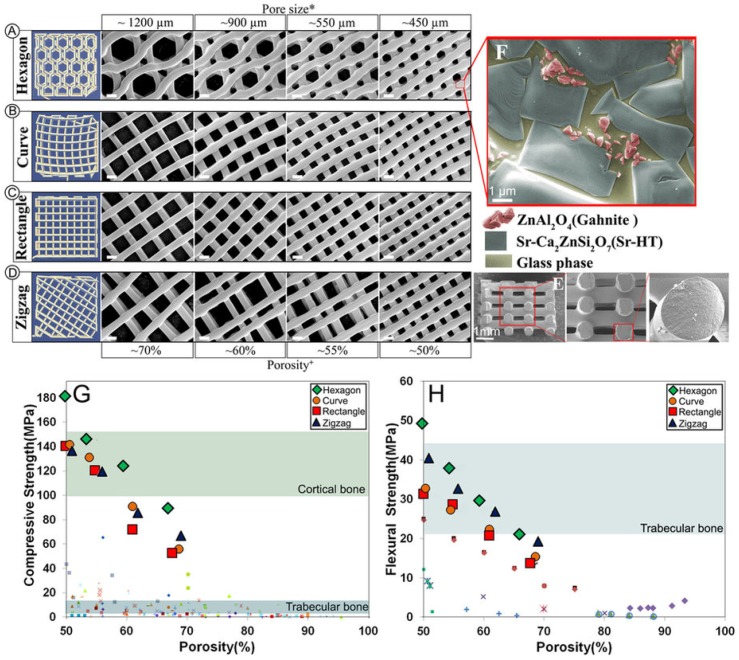
Sr-HT-Gahnite is a multiphasic ceramic produced by co-sintering of Sr-hardystonite with 15 wt% alumina. (**A**–**D**) Sr-HT-Gahnite scaffolds with a range of controlled geometries could be fabricated by 3D printing, all of which exhibited a unique microstructure featuring (**E**) solid struts and (**F**) three different phases. The (**G**) compressive strength and (**H**) flexural strength of 3D printed Sr-HT-Gahnite scaffolds greatly exceeded the values exhibited by other bioactive ceramic scaffolds at comparative porosities, and were within the ranges of values reported for human bone [66]. Reproduced by permission of the Nature Publishing Group, Copyright © 2016.

DCSC-inorganic composites can be fabricated by mixing and co-sintering DCSCs with another inorganic precursor, resulting in materials with unique microstructures and enhanced mechanical properties. Some studies have produced DCSC-inorganic composites with improved mechanical behaviour due to the formation of an additional glassy phase at the grain boundaries. For example, naturally-derived hydroxyapatite sintered with 10 wt % hardystonite resulted in increased density, with the formation of glass bonds at the boundaries of hydroxyapatite and hardystonite which contributed to improved compaction behaviour [112]. The co-sintering of hardystonite with calcium silicate could produce highly porous scaffolds with porosity exceeding 86%, which exhibited a glassy phase at the grain boundaries that was responsible for higher microstructural density and improved compressive strength compared to both α-CS and hardystonite scaffolds [28]. Notably, the co-sintering of Sr-hardystonite with 15 wt % alumina produced a multiphasic ceramic with a unique microstructure, consisting of crystalline Sr-hardystonite grains with a wetting glass phase at the grain boundaries, embedded within which were submicron gahnite (ZnAl_2_O_4_) crystals [113]. This microstructure was responsible for significant enhancements in mechanical properties, as the glass phase prevented crack propagation along the grain boundaries, and the submicron crystals also minimised microscopic crack propagation within the glass phase by acting as crack deflectors. When fabricated into scaffolds using the polymer sponge sacrificial template method, this ceramic (named Sr-HT-Gahnite) exhibited high compressive strength (4.1 MPa at 85% porosity) and fracture toughness (7.4–10 MPa·m^1/2^) which exceeded the mechanical properties of most calcium phosphates, bioactive glasses and DCSCs at comparative porosities [113,114]. By utilising 3D printing for the fabrication of Sr-HT-Gahnite scaffolds, a range of controlled pore geometries could be obtained which influenced the mechanical properties of the scaffold. Hexagonal pores were found to give the highest compressive strength of 90 MPa at 70% porosity, which was within the reported range of values for cortical bone (Figure 7) [66]. Sr-HT-Gahnite scaffolds also exhibited high bioactivity and osteogenic ability both in vitro and in vivo, which supported their development as an effective bone substitute [113,115].

Other studies have incorporated nano-sized components into DCSCs to produce DCSC-inorganic composites with enhanced strength and toughness. Porous diopside scaffolds reinforced with 2 wt % multi-walled carbon nanotubes (MWCNTs) exhibited significant improvements in compressive strength (10 MPa to 20 MPa) and fracture toughness (1.5 MPa·m^1/2^ to 3.2 MPa·m^1/2^), and the reinforcing mechanisms were identified as MWCNT crack deflection, crack bridging and pull-out [116]. Akermanite scaffolds reinforced with 1 wt % boron nitride nanosheets (BNN) also showed substantial improvements in compressive strength (6 MPa to 12 MPa) and fracture toughness (1.9 MPa·m^1/2^ to 2.3 MPa·m^1/2^), due to BNN wrapping of the grains within the akermanite matrix and sheet pull-out [117]. The incorporation of 5 wt % titania nanoparticles into akermanite scaffolds similarly improved the compressive strength (3.5 MPa to 22.9 MPa) and fracture toughness (1.8 MPa·m^1/2^ to 2.3 MPa·m^1/2^), through mechanisms of grain size refinement, crack deflection, and transition from intergranular to transgranular fracture mode [118]. Importantly, these studies also showed that the incorporation of nano-sized components into DCSCs had no negative effects on in vitro cell viability [116,117,118]. On the other hand, DCSCs have been used as the reinforcing phase to improve the biological activity of bioinert ceramics. For example, the incorporation of diopside microparticles into alumina at 1 wt % and 20 wt % induced surface apatite formation in SBF, as well as dramatic increases in flexural strength (130 MPa to 427 MPa) and fracture toughness (3.1 MPa·m^1/2^ to 4.3 MPa·m^1/2^) for 1 wt% diopside compared to unmodified alumina [119]. Similarly, the incorporation of 10 wt% diopside microparticles into hydroxyapatite induced surface apatite formation in SBF, alongside increases in flexural strength (27 MPa to 80 MPa) and fracture toughness (0.9 MPa·m^1/2^ to 1.2 MPa·m^1/2^) [120].

### 8.2. Polymer-DCSC Composites

**Figure 8:**
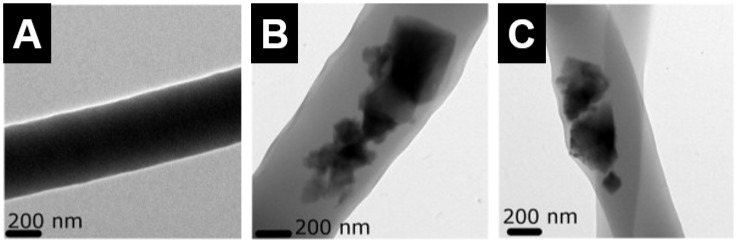
Transmission electron microscope images of electrospun nanofibres of (**A**) polycaprolactone (PCL); (**B**) PCL-hydroxyapatite (containing 40 wt % hydroxyapatite particles); and (**C**) PCL-hardystonite (containing 40 wt % hardystonite particles). The PCL-hardystonite nanofibres exhibited the highest tensile strength [122]. Adapted by permission of Elsevier, Copyright © 2013.

Polymer-DCSC composites have been fabricated by incorporating DCSCs as a reinforcing phase into polymer matrices. The DCSCs, which are often incorporated as micro- or nano-sized particles, can enhance the biological properties of bioinert polymers due to their ability to release bioactive ions, while simultaneously improving the mechanical properties of the polymer matrix. Several common synthetic polymers have been used to form composites with DCSCs, including polycaprolactone (PCL), poly(glycolic acid) (PGA) and poly(lactic-co-glycolic acid) (PLGA). Porous PCL-akermanite scaffolds (~90% porosity, ~100 µm pore size) were fabricated by loading akermanite powder into the PCL matrix at different weight ratios [121]. The highest compressive strength was attained at 25 wt % loading of akermanite particles (~10 MPa), compared to the other groups (50 wt % loaded, 75 wt % loaded, and PCL control) which all exhibited similar strengths (~4.3 MPa). Nevertheless, scaffolds loaded with 75 wt % akermanite particles showed the best biological activity when tested using human adipose-derived stem cells (hASCs), which enhanced cell viability and osteogenic gene expression (osteocalcin and alkaline phosphatase (ALP)) while reducing interleukin (IL)-6 expression. Hardystonite and hydroxyapatite powders were mixed with PCL to produce composite nanofibres by electrospinning (Figure 8) [122]. PCL-hardystonite nanofibres containing 40 wt % hardystonite particles showed increase in tensile strength (~10 MPa) compared to the PCL control (~6 MPa). This increase was more significant than that exhibited by PCL-hydroxyapatite nanofibres containing 40 wt % hydroxyapatite particles (~8 MPa), which was attributed to reduced agglomeration and better dispersion of the hardystonite particles in the PCL matrix. In addition, the PCL-hardystonite nanofibres promoted ALP activity and calcium mineralisation of murine adipose-derived stem cells to a greater extent than PCL-hydroxyapatite and PCL nanofibres. An injectable composite fabricated by incorporating 10 vol % baghdadite particles into PCL reached a peak flexural strength of 30 MPa, compared to 24 MPa for the PCL control [86]. In addition, the inclusion of baghdadite enhanced the radiopacity of the composite, as well as the proliferation and osteogenic gene expression (Runx2, osteocalcin, osteopontin) of primary human osteoblasts. Porous PGA scaffolds containing 10 wt % diopside microparticles and fabricated by selective laser sintering exhibited significant increases in compressive strength (10 MPa to 29 MPa), as well as enhanced apatite formation in SBF and reduced acidification effect of PGA degradation which improved the proliferation of MG-63 cells [123]. Similarly, akermanite powder was found to neutralise the acidic products from polymer degradation when incorporated into PLGA beads to form a drug delivery system [124]. PLGA beads containing various weight ratios of akermanite enhanced the proliferation and ALP activity of human bone marrow-derived mesenchymal stem cells, while maximum compressive modulus was achieved at 33 wt% incorporation of akermanite. 

DCSCs have also been used to modulate the properties of naturally-derived polymers, including silk fibroin, chitosan and gelatin, with the greatest changes often observed in the physical and structural properties of the resulting composites. Nano-sized diopside powder incorporated at 20–40 wt % into a silk fibroin matrix resulted in decreased porosity, increased compressive strength (~0.1 MPa to ~0.4 MPa) and modulus (~1 MPa to ~4 MPa), and slightly improved proliferation of MC3T3-E1 cells [125]. Diopside particles incorporated into a chitosan matrix were found to reduce the water retention capacity of the composite, while promoting the expression of osteogenic markers (ALP and collagen type I) in MG-63 cells [126]. The incorporation of akermanite as nano-sized powder into gelatin scaffolds was found to modulate the pore structure, mechanical properties and degradation behaviour depending on the weight ratio of akermanite [127]. In all of these studies, changes in porosity and water retention capacity of the natural polymer-DCSC composite is thought to be the result of variations in gelation and cross-linking between the polymer chains during scaffold formation due to ion release from the DCSCs. 

The future development of polymer-DCSC composites can draw benefits from recent advances in the fabrication and optimisation of biocompatible polymer blends, which can be used as potential matrices for the formation of new composites. Some examples include PCL/poly(lactic acid) (PLA) [128], poly(hydroxybutyrate-co-hydroxyvalerate) (PHBV)/poly(l-lactic acid) (PLLA) [129], and PCL/gelatin [130], which have been fabricated through advanced techniques such as additive manufacturing, emulsion freezing/freeze-drying, and electrospinning. An important consideration in the development of new polymer-DCSC composites is the effect of ceramic particle concentration on the physicochemical properties of the polymer matrix. For polymer-ceramic composites containing micro- or nano-sized ceramic particles, optimal mechanical properties are typically obtained at a threshold particle concentration, above which the mechanical properties of the composite are negatively affected due to issues such as agglomeration and particle-to-particle interactions [131,132,133]. This threshold concentration is often determined experimentally, although some recent studies have developed mathematical models to predict the effects of varying ceramic particle concentrations on the mechanical properties of polymer-ceramic composites [134,135].

### 8.3. Coating of DCSC Scaffolds

**Figure 9:**
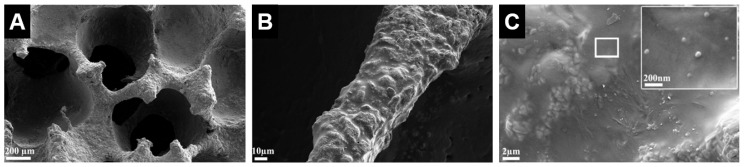
Scanning electron microscope images of baghdadite scaffolds reinforced with a PCL coating containing bioactive glass nanoparticles showed that (**A**) the scaffolds maintained a highly porous structure after coating; (**B**) the coated struts had a smooth surface with absence of visible cracks or pores; and (**C**) the coating was homogeneous with evenly dispersed bioactive glass nanoparticles within the PCL [71]. Adapted by permission of Elsevier, Copyright © 2012.

A prominent issue encountered with the majority of ceramic scaffolds, including those composed of DCSCs, is their inherent brittleness and poor ability to sustain the high compressive stresses present in load-bearing bone defects. The low compressive strength and fracture toughness of ceramic scaffolds are exacerbated at the high porosities which are desirable for encouraging bone regeneration. The brittle failure of ceramic scaffolds under load is often the result of crack propagation from microscopic defects, such as microcracks and micropores, on the surface of scaffold struts which act as stress concentration points [136]. This can give rise to large amounts of loose ceramic particles at the defect site, and induce an inflammatory response in a similar manner as prosthetic wear debris [137]. A simple and commonly employed method for improving the mechanical properties of ceramic scaffolds is to fill the existing surface defects by coating the scaffold with a thin polymer layer. The polymer coating functions in crack bridging and energy dissipation, thereby reducing scaffold brittleness by lowering the chance of crack propagation under load [138]. The polymer coating is applied by first dissolving the polymer in a suitable solvent, then dipping the ceramic scaffold and subsequently evaporating the solvent to leave a thin layer of the polymer on the scaffold surface. This method of reinforcement has been applied to several DCSC scaffolds to improve their mechanical properties. For example, akermanite scaffolds coated with poly(d,l-lactic acid) (PDLLA) showed significant increase in compressive strength that was proportional to the concentration of the PDLLA solution used for coating [139]. At 70% porosity, PDLLA-coated akermanite scaffolds showed compressive strength of ~4 MPa, compared to ~2 MPa for uncoated scaffolds. The PDLLA-coated akermanite scaffolds also exhibited a reduced degradation rate which led to enhanced proliferation of MC3T3-E1 cells. The improved biological activity of coated scaffolds was thought to be the result of smaller pH changes and more controlled release of Ca, Si and Mg in the surrounding environment due to the masking effect of the coating. In another study, baghdadite scaffolds were reinforced using a modified PCL coating containing bioactive glass nanoparticles (nBG), where the nBG were included to improve the biological activity of the coating (Figure 9) [71]. The coated scaffolds achieved a compressive strength of 1.1 MPa and failure strain of 7%, compared to values of 0.2 MPa and 0.5% for unmodified scaffolds. In both small (rabbit) [71] and large (sheep) [110] animal models, baghdadite scaffolds with the nanocomposite PCL-nBG coating achieved favourable outcomes of bone defect repair which were comparable to unmodified scaffolds, and the coating was thought to have an important role in maintaining initial mechanical stability after implantation.

### 8.4. DCSC-Coated Metal Implants

**Figure 10:**
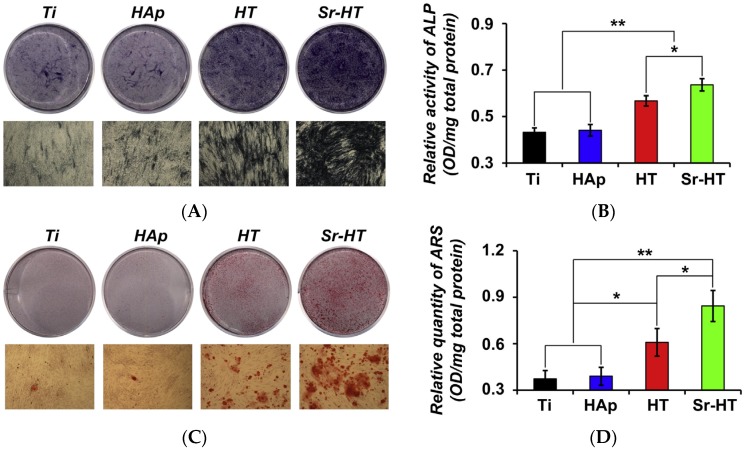
Extracts of Ti-6Al-4V samples coated with Sr-hardystonite enhanced the osteogenic activity of bone marrow-derived mesenchymal stem cells compared to extracts of hardystonite-coated and hydroxyapatite-coated samples, as shown by (**A**) alkaline phosphatase staining; (**B**) quantitative analysis of alkaline phosphatase activity; (**C**) Alizarin Red S staining; and (**D**) quantitative analysis of calcium deposition activity [141]. (* *p* < 0.05; ** *p* < 0.01). Reproduced by permission of Elsevier, Copyright © 2013.

Titanium and its alloys (such as Ti-6Al-4V) are the most commonly used materials for hip and knee implants, as well as bone plates and screws in orthopaedic and dental applications due to their biocompatibility, good mechanical properties and corrosion resistance [140]. However, titanium implants in clinical use are often unable to achieve sufficient osseointegration to establish a structural and functional connection with the surrounding bone in the long-term. Consequent micro-movement at the implant-bone interface can result in inadequate implant fixation and fibrous tissue formation, ultimately leading to aseptic loosening and premature implant failure [141]. Hydroxyapatite coatings are now commonly used on metal implants in hip and knee replacements to improve osseointegration due to their chemical similarity to the mineral component of bone. However, hydroxyapatite coatings are prone to delamination and fragmentation, due to unresolved issues such as poor coating adhesion to the underlying metal and mismatch in thermal expansion coefficient between the coating and implant [142]. Due to these existing problems, the application of DCSCs as orthopaedic implant coatings has been explored. Along with calcium silicate, a range of DCSCs have been coated onto titanium alloys and exhibited significant increases in bonding strength compared to hydroxyapatite coatings. The bonding strength of DCSC coatings to the titanium substrate was generally within the range of 25–45 MPa, including akermanite (42.2 MPa) [143], diopside (32.5 MPa) [144], sphene (33.2 MPa) [145], baghdadite (28 MPa) [146], hardystonite (~26 MPa) [141], and Sr-hardystonite (~35 MPa) [141]. In comparison, calcium silicate coatings on titanium showed bonding strength of 24–43 MPa [147,148], while hydroxyapatite coatings were within the range of 10–20 MPa [146,149]. The high bonding strengths of DCSC and calcium silicate coatings are favourable for maintaining implant stability from a mechanical perspective. However, considering that the degradation behaviour of ceramic coatings affects the ion release and pH of the surrounding environment, which directly influence cellular interactions with the implant, the more chemically stable DCSC coatings are preferred over calcium silicate coatings for long-term implantation. In particular, DCSCs which exhibit slow degradation may be well suited for applications as implant coatings, such as diopside [36,70], sphene [40], hardystonite [25] and gehlenite [34]. Sr-hardystonite is another favourable candidate, as demonstrated by enhanced in vitro attachment and osteogenic activity of bone marrow-derived mesenchymal stem cells when coated onto a titanium substrate compared to hardystonite-coated, hydroxyapatite-coated and uncoated samples (Figure 10) [141]. In addition, samples coated with Sr-hardystonite achieved the best outcomes of in vivo osseointegration in a canine femur model.

Recent work on developing DCSCs as implant coatings has applied the technology on implantable magnesium alloys, in order to reduce their fast corrosion rate and subsequent alkalinity which negatively affect osseointegration and viability of the bone tissue surrounding the implant. Hardystonite coating on a Mg-Ca-Zn alloy was found to reduce the corrosion rate and alkalinity (pH ~10.5 to ~8.5), and enhance the in vitro viability of MC3T3-E1 cells compared to the uncoated control [150]. AZ91 magnesium alloy coated with diopside showed significant reductions in corrosion rate, magnesium ion release, and alkalinity (pH ~10.5 to ~8.5) [151], as well as enhanced in vitro viability of L-929 fibroblast cells [152] and in vivo bone formation on the implant surface in the greater trochanter defect of a rabbit model [153]. Similar results were obtained when the AZ91 alloy was coated with akermanite [154,155,156].

## 9. Conclusions and Future Perspectives

DCSCs are a novel class of bioactive ceramics with a unique set of properties, which make them suitable for use as synthetic bone substitutes with the potential to produce improved outcomes compared to existing ceramic materials. The *x*CaO–*y*SiO_2_ system is highly versatile, enabling doping with a range of ions and oxides to form different DCSCs with tailored properties depending on (1) stoichiometric composition; (2) fabrication method; and (3) the role of the DCSC in composite systems. The physicochemical properties of DCSCs, such as mechanical behaviour, degradation and ion release characteristics, and radiopacity can be optimised to produce enhanced in vitro cell interactions and in vivo bone regeneration outcomes. A number of DCSCs and DCSC-based composites already display properties which satisfy the structural, mechanical and biological requirements for bone regeneration at load-bearing defect sites, such as akermanite, baghdadite, Sr-hardystonite and Sr-HT-Gahnite. In order to propel the translation of DCSCs into clinical use as solid or scaffold implants, composites, and coatings, future investigations should focus on understanding the long-term biological interactions with DCSCs in an in vivo setting. DCSC-based implants intended for clinical use should be tested in animal models with bone defects which are of similar structure and characteristics as those encountered in humans. When evaluating the outcomes, it will be important to clarify the interactions of bone-related cells with DCSCs and the pathways involved in generating an enhanced regenerative response. Long-term studies will be necessary to monitor implant degradation and bone remodelling over time and ensure the restoration of original bone architecture. In addition, the antibacterial activity of certain DCSCs can be exploited to produce improved implants which minimise the risk of infection. Such investigations will accelerate the development of DCSCs as the next generation of synthetic bone substitutes. 

## Figures and Tables

**Table 1:** Stoichiometric formula, reported fabrication method and heat treatment for a range of doped calcium silicate ceramics (DCSCs), as well as α- and β-calcium silicate. Calcination temperature is reported if higher sintering temperature is not provided.

Ceramic	Stoichiometric Formula	Fabrication Method and Heat Treatment	Ref.
**α-calcium silicate (α-CS) (pseudowollastonite)**	CaO–SiO_2_	Chemical precipitation, sintered at 1250 °C for 3 h	[27]
**β-calcium silicate (β-CS)**	CaO–SiO_2_	Chemical precipitation, sintered at 1100 °C for 3 h Chemical precipitation, sintered at 1090 °C for 2 h	[27] [30]
**Sr-α-CaSiO_3_ (Sr-α-CS)**	*x*SrO–(1 − *x*)CaO–SiO_2_; *x* = 0.01~0.10	Chemical precipitation, sintered at 1250 °C for 3 h	[31]
**Sr-β-CaSiO_3_ (Sr-β-CS)**	*x*SrO–(1 − *x*)CaO–SiO_2_; *x* = 0.10	Chemical precipitation, sintered at 1090 °C for 2 h	[30]
**Cu-β-CaSiO_3_ (Cu-CS)**	*x*CuO–(1 − *x*)CaO–SiO_2_; *x* = 0.025	Chemical precipitation, calcined at 900 °C for 2 h	[32]
**Akermanite (AK)**	2CaO–MgO–2SiO_2_	Sol-gel, sintered at 1370 °C for 6 h	[37]
**Co-Akermanite (Co-AK)**	2CaO–CoO–2SiO_2_	Sol-gel, sintered at 1200 °C for 3 h	[42]
**Diopside (DS)**	CaO–MgO–2SiO_2_	Co-precipitation, sintered at 1300 °C for 2 h	[36]
**Bredigite (BD)**	7CaO–4SiO_2_–MgO	Sol-gel, sintered at 1350 °C for 8 h	[38]
**Hardystonite (HT)**	2CaO–ZnO–2SiO_2_	Sol-gel, sintered at 1350 °C for 5 h Sol-gel, sintered at 1250 °C for 3 h	[39] [25]
**Sr-hardystonite (Sr-HT)**	*x*SrO–(2 − *x*)CaO–ZnO–2SiO_2_; *x* = 0.10	Sol-gel, sintered at 1250 °C for 3 h	[25]
**Sphene (Sph)**	CaO–TiO_2_–SiO_2_	Sol-gel, sintered at 1280 °C, time not reported	[40]
**Baghdadite (Bag)**	3CaO–ZrO_2_–2SiO_2_	Sol-gel, sintered at 1400 °C for 3 h Solid-state sintering at 1400 °C for 3 h	[33] [51]
**Sr-Bag (Sr-Bag)**	*x*SrO–(3 − *x*)CaO–ZrO_2_–2SiO_2_, *x* = 0.1, 0.75	Solid-state sintering at 1400 °C for 3 h	[41]
**Cuprorivaite (Cup)**	CaO–CuO–4SiO_2_	Sol-gel, calcined at 1000 °C	[35]
**Gehlenite (GLN)**	2CaO–Al_2_O_3_–SiO_2_	Solid-state sintering at 1400 °C for 3 h	[34]

**Table 2:** Mechanical properties (Young’s modulus, mechanical strength, and fracture toughness) of a range of DCSCs, as well as α- and β-calcium silicate. Values for Bioglass 45S5, hydroxyapatite, β-tricalcium phosphate (β-TCP), and biphasic calcium phosphate (BCP), as well as cortical and cancellous bone are included for comparison. Specimens with porosities <20% were considered ‘dense’, while those with porosities >50% were considered ‘scaffold’.

Ceramic	Porosity (%)	Young’s Modulus (GPa)	Mechanical Strength (MPa)	Fracture Toughness (MPa·m^1/2^)	Ref.
**α-calcium silicate**	15.5	NR	39.7^B^	NR	[27]
	82.2^PSST^	~0.012	0.3^C^	NR	[29]
	~89^PSST^	NR	0.03^C^	NR	[28]
**β-calcium silicate**	18.6	NR	65.9^B^	NR	[27]
**Sr-α-CaSiO_3_**	*No mechanical property evaluation*				
**Sr-β-CaSiO_3_**	*No mechanical property evaluation*				
**Cu-β-CaSiO_3_**	*No mechanical property evaluation*				
**Akermanite**	10.4	42	176.2^B^	1.83	[68]
	63.5^PSST^ 81.7^PSST^ 90.3^PSST^	NR	1.13^C^ 0.79^C^ 0.53^C^	NR	[69]
	57.9^SLS^	NR	5.9^C^	1.72	[63]
	53^DIW^	~0.5	71^C^	NR	[64]
**Co-akermanite**	*No mechanical property evaluation*				
**Diopside**	NR (dense)	170	300^B^	3.5	[52]
	75^PSST^ 82^PSST^	0.07 0.01	1.4^C^ 0.5^C^	NR	[70]
**Bredigite**	5.8 ~90^PSST^	43 NR	156^B^ 0.233^C^	1.57 NR	[38]
**Hardystonite**	17.4	NR	136.4^B^	1.24	[39]
	77.5^PSST^	NR	1.99 ± 0.45^C^	NR	[25]
	~89^PSST^	NR	0.06	NR	[28]
	74^DIW^	NR	1.6 ± 0.3^C^	NR	[65]
**Sr-hardystonite**	78^PSST^	NR	2.16 ± 0.52^C^	NR	[25]
**Sphene**	*No mechanical property evaluation*				
**Baghdadite**	0.5	120	98^B^	1.3	[51]
	2.8	NR	168^B^	1.2	[41]
	~88^PSST^	~0.0153	~0.27^C^	NR	[71]
**Sr-Baghdadite**	3.4	NR	162^B^	1.3	[41]
**Cuprorivaite**	*No mechanical property evaluation*				
**Gehlenite**	0.3	112	162^B^; 403^C^	2.7	[34]
**Bioglass 45S5**	Dense	35	42	NR	[72]
**Bioglass 45S5-derived scaffold**	86–94	NR	0.3–1.2^B^; 0.05–0.45^C^	NR	[73]
**Hydroxyapatite (HA)**	NR (dense)	47	110^B^	1.1	[52]
	<0.8	80–110	100–160^B^; 500^C^	1.0	[72]
	2.2–7.0	87–97	84–113^B^	0.69–0.96	[74]
**β-Tricalcium phosphate (β-TCP)**	<0.3	33–90	140–154^B^; 460–687^C^	NR	[72]
	0.6~1.4	87–95	118–133^B^	1.14–1.30	[74]
**Biphasic calcium phosphate (BCP)**	~88^PSST^	0.0105	0.12^C^	NR	[71]
**Cortical bone**	5–13	12–18	50–150^B^; 130–180^C^	2–12	[7,13,17]
**Cancellous bone**	30–90	0.1–0.5	10–20^B^; 4–12^C^	0.1–0.8	[7,13,17]

NR: not reported; PSST: polymer sponge sacrificial template; DIW: direct ink writing; SLS: selective laser sintering; B: bending; C: compression.

**Table 3:** Summary of in vitro degradation studies for calcium silicate and a range of DCSCs in aqueous media. All ion release values are reported in parts per million (ppm), where ppm = mM × A (atomic mass) for concentrations reported in mM. Background ion concentration was subtracted if background values were provided. Numbers in brackets indicate values obtained for the α-calcium silicate (α-CS) control in the same experiment.

Ceramic	Morphology and Concentration	Surrounding Aqueous Media	Weight Loss after 7 Days, (α-CS Value)	pH of Media after 7 Days, (α-CS Value)	Apatite Formation in SBF	Total ion Release in Media after 7 Days ^unless otherwise stated^, (α-CS Value)	Ref.
**β-calcium silicate**	Solid disks, ratio of disk to media not reported	CM	NR	NR	Yes	Ca: ~160 ppm, (~120 ppm)	[84]
						Si: ~90 ppm, (~80 ppm)	
**Sr-α-CaSiO_3_**	Solid disks, at 0.1 cm^2^/mL	SBF	5% at 2.5 mol Sr, (7%)	8.3, (8.4)	Yes	Ca: ~260 ppm, (~310 ppm)	[31]
						Si: ~65 ppm, (~98 ppm)	
						Sr: ~2.6 ppm	
			7% at 10 mol Sr, (7%)	8.0, (8.4)	Yes	Ca: ~260 ppm, (~310 ppm)	[31]
						Si: ~85 ppm, (~85 ppm)	
						Sr: ~7.9 ppm	
**Sr-β-CaSiO_3_**	*No degradation evaluation of sintered disks/scaffolds*						
**Cu-β-CaSiO_3_**	*No degradation evaluation of sintered disks/scaffolds*						
**Akermanite**	Solid disks, at 0.1 cm^2^/mL	SBF	NR	7.3	Yes	Ca: ~240 ppm	[37]
						Si: ~62 ppm	
						Mg: ~121 ppm	
	Solid disks, at 0.15 mm^3^/mL	Tris-HCl	2.50%	NR	Yes	NR	[36]
	Solid disks in 48-well plate	CM	NR	NR		Ca: ~95 ppm	[36]
						Si: ~26 ppm	
						Mg: ~30 ppm	
	Solid disks, 10 mm diameter in 1 mL solution	CM	NR	NR		Ca: ~100 ppm	[85]
						Si: ~100 ppm	
						Mg: ~195 ppm	
	Porous scaffolds at 5 mg/mL	Ringer’s solution	7%	NR	Yes	Cannot deduce concentration as volume of samples was not reported	[69]
**Co-akermanite**	*No degradation evaluation of sintered disks/scaffolds*						
**Diopside**	Solid disks, at 0.15 mm^3^/mL	Tris-HCl	0.50%	NR	Yes	NR	[36]
	Solid disks in 48-well plate	CM	NR	NR		Ca: ~87 ppm	[36]
						Si: ~70 ppm	
						Mg: ~20 ppm	
	Porous scaffolds at 5 mg/mL	SBF	1.00%	7.5	Yes	Si: ~150 ppm	[70]
**Bredigite**	Solid disks, at 0.15 mm^3^/mL	Tris-HCl	5%	NR	Yes	NR	[36]
	Solid disks in 48-well plate	CM	NR	NR		Ca: ~70 ppm	[36]
						Si: ~32 ppm	
						Mg: ~20 ppm	
**Hardystonite**	Solid disks, at 0.1 cm^2^/mL	SBF	NR	7.5	No	Ca: ~100 ppm^14 days^, (~600 ppm)	[81]
						Si: ~33 ppm^14 days^, (~75 ppm)	
						Zn: ~0.4 ppm^14 days^	
	Porous scaffolds at 5 mg/mL	SBF	0.7%, (8%)	7.2, (8.6)	No	Ca: ~16 ppm, (340 ppm)	[25]
						Si: ~6 ppm, (98 ppm)	
						Zn: ~0.004 ppm	
	Porous scaffolds (7 × 7 × 7 mm^3^) in 15 mL	Tris-HCl	~3%, (~11%)	7.5, (8.2)	NR	Ca: 22 ppm, (144 ppm)	[28]
						Si: 5 ppm, (19 ppm)	
						Zn: 1 ppm	
**Sr-hardystonite**	Porous scaffolds at 5 mg/mL	SBF	1.2%, (8%)	7.7, (8.6)	Yes	Ca: ~40 ppm, (340 ppm)	[25]
						Si: ~11 ppm, (98 ppm)	
						Zn: ~0.0005 ppm	
						Sr: ~0.6 ppm	
**Sphene**	Solid disks, at 0.1 cm^2^/mL	SBF	~0%, (7%)	~7.7, (~8.4)	No	Ca: ~20 ppm, (~310 ppm)	[40]
						Si: 0 ppm, (~98 ppm)	
						Ti: 0 ppm	
**Baghdadite**	Solid disks, ratio of disk to media not reported	CM	NR	7.5, (8.1)	Yes	Ca: ~370 ppm, (~384 ppm)	[33]
						Si: ~44 ppm, (~49 ppm)	
						Zr: 0 ppm	
	Porous scaffolds, 150 mg/L	SBF	9%	8	Yes	Ca: ~200 ppm	[71]
						Si: ~32 ppm	
						Zr: 0.0005 ppm	
**Sr-Baghdadite**	*No degradation evaluation of sintered disks/scaffolds*						
**Cuprorivaite**	*No degradation evaluation of sintered disks/scaffolds*						
**Gehlenite**	Solid disks, at 0.1 mm^2^/mL	SBF	~0%	~7.4	No	Ca: ~45 ppm^9 days, SBF^	[34]
		Tris-HCl	~1%	~7.4		Si: ~5 ppm^9 days, SBF^	
		Citric acid	~7%	~4		Al: ~10 ppm^9 days, SBF^	

NR: not reported; SBF: simulated body fluid; CM: cell culture media.

**Table 4:** X-ray mass attenuation coefficient (XMAC) for a range of materials, including DCSCs, in ascending order, calculated using Equation (1) at 20 keV X-ray energy.

Ceramic	XMAC at 20 keV (Dense Material)
Cortical bone	4.00
Bioglass 45S5	4.09
Diopside	4.27
Gehlenite	5.31
Akermanite	5.36
α-, β-CaSiO_3_	5.94
Hydroxyapatite	6.38
Tricalcium phosphate	6.49
Bredigite	6.62
Sphene	7.53
Cu-β-CaSiO_3_ (2.5 mol % substitution of Ca)	9.26
Cuprorivaite	9.54
Sr-α-, β- CaSiO_3_ (10 mol % substitution of Ca)	9.90
Co-akermanite	9.91
Hardystonite	12.96
Sr-hardystonite (5 mol % substitution of Ca)	13.61
Baghdadite	20.76
Sr-Baghdadite (25 mol % substitution of Ca)	21.74

**Table 5:** Summary of in vitro studies on DCSCs performed using a range of cell types and material morphologies (powder extracts, dense disks and porous scaffolds).

Ceramic	Cell Type	Ceramic Morphology	Main Findings	Ref.
**Sr-α-CaSiO_3_** **(Sr-α-CS)**	Human bone-derived cells	Powder ionic extract	Sr ions in Sr-α-CS extract enhanced cell proliferation at lower Ca and Si concentrations, compared to α-CS extracts with no Sr	[31]
**Sr-β-CaSiO_3_** **(Sr-β-CS)**	Ovariectomised rat bone marrow-derived stem cells	Powder ionic extract	Enhanced cell proliferation, ALP activity, and osteogenic gene expression (Runx2, BSP, OC, VEGF, OPG/RANKL ratio) in Sr-β-CS extract (6.25 mg/mL) compared to β-CS extract	[30]
	Human umbilical vein endothelial cells	Powder ionic extract	Enhanced cell proliferation, angiogenic gene expression (VEGF, KDR), and in vitro angiogenesis in Sr-β-CS extract (3.1~12.5 mg/mL) compared to β-CS extract	[30]
**Cu-β-CaSiO_3_** **(Cu-β-CS)**	Human umbilical vein endothelial cells	Powder ionic extract	No difference in cell proliferation between β-CS and Cu-β-CS extracts; enhanced angiogenic gene expression (VEGF, KDR, HIF-1α) and in vitro angiogenesis in Sr-β-CS extract (3.1~12.5 mg/mL) compared to β-CS extract	[32]
**Akermanite** **(AK)**	Human bone marrow-derived stromal cells	Powder ionic extract	Enhanced proliferation, ALP activity, and osteogenic gene expression (OC, OPN) in AK extract (0.78 mg/mL) compared to β-TCP control	[98]
	Human bone marrow-derived stromal cells	Direct seeding on dense ceramic disks	Enhanced proliferation, ALP activity, and osteogenic gene expression (ALP, BSP, OPN) on AK disk compared to β-TCP control	[99]
	Calf bone marrow stromal cells	Direct seeding on porous scaffold	Cells attached on AK scaffold; no significant difference in cell proliferation and ALP activity on AK scaffold compared to tissue culture plastic	[69]
	Human periodontal ligament cells	Direct seeding on dense ceramic disks	Enhanced attachment, proliferation, and osteogenic gene expression (OPN, DMP-1, OC) on AK disk compared to β-TCP control	[85]
	Human adipose-derived stem cells	Powder ionic extract	Slight inhibition of proliferation at high AK extract concentrations (25~100 mg/mL) compared to no AK extract control; significantly enhanced ALP activity, mineralisation, and OCN synthesis of cells in AK extract (25~50 mg/mL) compared to no extract control; enhanced osteogenic gene expression (Cbfα1, ALP, OCN), but reduced Col1 expression compared to no extract control; ERK pathway implicated in stimulation of osteogenic differentiation	[94]
	Human induced pluripotent stem cells	Powder ionic extract	AK extracts had no cytotoxic effects or effects on cell stemness; enhanced ALP activity, mineralisation, and osteogenic gene expression (ALP, BMP-2, Col1, OCN, Runx2) compared to culture medium without AK extract, with optimal extract concentration at 1.56 mg/mL	[97]
	Rat bone marrow-derived stem cells	Powder ionic extract	Enhanced proliferation, ALP activity, osteogenic (Runx2, BMP-2, BSP, OPN, OC, OPG/RANKL) and angiogenic (VEGF, ANG-1) gene expression, and inhibited TNF-α expression of cells in AK extract (12.5 mg/mL) compared to β-TCP control; activated ERK, P38, AKT and STAT3 pathways	[100]
	Rat bone marrow macrophages	Powder ionic extract	Inhibited mature osteoclast formation and osteoclastogenesis (TRAP, cathepsin K, NFATcl) compared to β-TCP control	[100]
	Human bone marrow-derived mesenchymal stem cells	Powder ionic extract	Enhanced cell proliferation (at 0.78–3.1 mg/mL), ALP activity, and osteogenic gene expression (OPN, Col1) compared to β-TCP extract	[101]
	Human aortic endothelial cells	Powder ionic extract	Enhanced cell proliferation, nitric oxide synthesis, angiogenic gene expression (eNOs, KDR, FGFR1, ACVRL1), and in vitro angiogenesis in AK extract (3.1~12.5 mg/mL) compared to β-TCP extract and ceramic-free control	[101]
**Co-akermanite** **(Co-AK)**	Mouse osteoblast-like cells (MC3T3-E1)	Powder ionic extract	Inhibited cell proliferation in Co-AK extract (6.25–200 mg/mL); enhanced ALP activity in Co-AK extract of 0.78 mg/mL compared to β-CS	[42]
	Human umbilical vein endothelial cells	Powder ionic extract	Inhibited cell proliferation in Co-AK extract (50–200 mg/mL); enhanced angiogenic gene expression (VEGF, eNOs) and in vitro angiogenesis in Co-AK extract of 0.78 mg/mL compared to β-CS	[42]
**Diopside** **(DS)**	Human periodontal ligament cells and human bone marrow-derived mesenchymal stem cells	Powder ionic extract	Enhanced proliferation of hPDLCs at 100–200 mg/mL compared to β-TCP and hardystonite; enhanced OCN expression of hBMSCs at 50 mg/mL	[95]
	Human bone marrow derived-mesenchymal stem cells	Powder ionic extract	Enhanced cell proliferation (at 1.6 mg/mL), ALP activity, and osteogenic gene expression (OPN) compared to β-TCP extract	[101]
	Human aortic endothelial cells	Powder ionic extract	No significant difference in cell proliferation, nitric oxide synthesis, angiogenic gene expression (eNOs, KDR, FGFR1, ACVRL1), and in vitro angiogenesis compared to β-TCP extract and ceramic-free control	[101]
**Bredigite** **(BD)**	Human bone marrow-derived mesenchymal stem cells	Powder ionic extract	Enhanced cell proliferation (at 0.39–3.1 mg/mL), ALP activity, and osteogenic gene expression (OPN, Col1) compared to β-TCP extract	[101]
	Human aortic endothelial cells	Powder ionic extract	Enhanced cell proliferation, nitric oxide synthesis, angiogenic gene expression (eNOs, KDR, FGFR1, ACVRL1), and in vitro angiogenesis in BD extract (3.1~12.5 mg/mL) compared to β-TCP extract and ceramic-free control	[101]
	Human periodontal ligament cells	Powder ionic extract	Enhanced cell proliferation at 6.25–25 mg/mL compared to tissue culture plastic; enhanced ALP activity and osteogenic gene expression (ALP, OC, OPN, BSP, CAP, CEMP1) at 50 mg/mL compared to tissue culture plastic; shown to activate Wnt/β-catenin signalling pathway	[96]
**Hardystonite** **(HT)**	Human osteoblast-like cells	Direct seeding on dense ceramic disks	Cells adhered; significantly enhanced cell proliferation and ALP activity of cells on HT disks compared to α-CS	[81]
	Human bone marrow derived mesenchymal stem cells	Direct seeding on dense ceramic disks; indirect co-culture of cells and ceramic disk	Enhanced proliferation in indirect culture compared to β-TCP and tissue culture plastic, while proliferation rate was lower for direct seeding; higher ALP activity on HT compared to β-TCP; significantly higher osteogenic expression (Col1, ALP, OPN, BSP, OC) compared to β-TCP for direct seeding	[102]
	Human periodontal ligament cells and human bone marrow-derived mesenchymal stem cells	Powder ionic extract	Enhanced ALP expression of hBMSCs at 12.5 mg/mL compared to diopside and β-TCP; enhanced antibacterial effect against *E. faecalis* compared to β-TCP, comparable antibacterial effect with calcium hydroxide	[95]
	Primary human osteoblasts	Direct seeding on porous ceramic scaffolds	Enhanced cell attachment and BSP gene expression for cells seeded on HT compared to calcium silicate, while all other osteogenic genes tested (Runx2, OPN, OC, Col1, ALP) showed insignificant difference or reduced expression compared to calcium silicate	[28]
	Primary human osteoblasts	Direct seeding on porous ceramic scaffolds	Enhanced cell proliferation and ALP activity on HT scaffolds compared to β-TCP, and enhanced OPN gene expression compared to tissue culture plastic	[25]
**Sr-hardystonite** **(Sr-HT)**	Primary human osteoblasts	Direct seeding on porous ceramic scaffolds	Enhanced osteogenic gene expression (OC, BSP, OPN, Runx2) on Sr-HT scaffolds compared to hardystonite scaffolds and tissue culture plastic	[25]
**Sphene** **(Sph)**	Primary human bone-derived cells	Direct seeding on dense ceramic disks	Cells adhered; significantly enhanced cell proliferation and ALP activity of cells on hardystonite disks compared to α-CS	[40]
**Baghdadite** **(Bag)**	Primary human osteoblasts	Direct seeding on dense ceramic disks	Enhanced proliferation, ALP activity, and osteogenic expression (Col1, ALP, BSP, OC, RANKL, OPG) on Bag disks compared to α-CS	[33]
	Primary human monocytes	Direct seeding on dense ceramic disks	Bag disks supported osteoclast differentiation from monocytes as opposed to α-CS	[33]
	Human dermal microvascular endothelial cells	Direct seeding on dense ceramic disks	Bag disks supported endothelial cell attachment and enhanced expression of VE-cadherin as opposed to α-CS	[33]
	Primary human ostoblasts; adipose-derived stem cells	Direct seeding on dense ceramic disks; indirect co-culture	Bag disks showed enhanced osteogenic expression in HOBs (Runx2, BSP, OPN, OC) and ASCs (Runx2, OPN); Bag shown to modulate cross-talk between HOBs and ASCs via BMP-2 pathway	[103]
	Unactivated macrophages derived from primary human monocytes	Direct seeding on porous scaffold; indirect co-culture	Bag disks promoted upregulation of genes related to pro-remodelling M2c phenotype	[108]
	Human periodontal ligament cells	Direct seeding on dense ceramic disks; powdered extract	Enhanced ALP activity, upregulated cementogenic and osteogenic gene expression, and upregulated Wnt/β-catenin pathway-related genes compared to β-TCP for both direct and indirect culture methods	[109]
	Human osteoblasts	Direct seeding on dense ceramic disks	Enhanced attachment, proliferation, and ALP expression of cells on Bag disks compared to α-CS	[41]
**Sr-Baghdadite** **(Sr-Bag)**	Human osteoblasts	Direct seeding on dense ceramic disks	Enhanced attachment, proliferation, and ALP expression of cells on Sr-baghdadite disks compared to α-CS, with optimal ALP expression at 0.7 mol % Sr substitution of calcium	[41]
**Cuprorivaite** **(Cup)**	Mouse osteoblast-like cells (MC3T3-E1)	Powder ionic extract	Cytotoxic at 25–200 mg/mL; inhibited ALP activity of cells cultured in 0.195–0.78 mg/mL Cup extract compared to β-CS	[35]
	Human umbilical vein endothelial cells	Powder ionic extract	Cytotoxic at 25–200 mg/mL; enhanced in vitro angiogenesis and VEGF expression of cells cultured in 0.39–0.78 mg/mL Cup extract compared to β-CS extract and copper extract; has antibacterial effects against *E. coli*	[35]
**Gehlenite** **(GLN)**	Primary human osteoblasts	Direct seeding on dense ceramic disks	Enhanced cell attachment, proliferation, and osteogenic gene expression (Runx2, OPN, BSP, OC) on GLN disks compared to biphasic calcium phosphate disks	[34]
	Mouse bone marrow macrophages	Direct seeding on dense ceramic disks	Promoted formation of TRAP-positive osteoclasts, and enhanced osteoclast attachment and polarisation	[34]

CS: calcium silicate; β-TCP: β-tricalcium phosphate; ALP: alkaline phosphatase; BSP: bone sialoprotein; Col1: collagen type I; OC: osteocalcin; OPN: osteopontin; VEGF: vascular endothelial growth factor; OPG: osteoprotegerin; RANKL: receptor activator of nuclear factor kappa-B ligand; HIF: hypoxia inducible factor; DMP: dentin matrix acidic phosphoprotein; TNF: tumour necrosis factor; ANG: angiopoietin; BMP: bone morphogenetic protein; TRAP: tartrate-resistant acid phosphatase; eNOS: endothelial nitric oxide synthase; FGFR1: fibroblast growth factor receptor 1; ACVRL1: activin A receptor like type 1; CAP: catabolite activator protein; CEMP1: cementum protein 1; hPDLCs: human periodontal ligament cells; hBMSCs: human bone marrow-derived mesenchymal stem cells; ASCs: adipose-derived stem cells; HOBs: human osteoblast-like cells.

**Table 6:** Summary of in vivo studies on DCSCs performed in a range of animal models using dense specimens or porous scaffolds.

Ceramic	Implant Morphology	Animal Model	Implantation Period	Main Findings	Ref.
**Sr-β-CaSiO_3_** **(Sr-β-CS)**	Porous scaffolds	Ovariectomised rat calvarial defects	4 weeks	µ-CT analysis showed higher bone mineral density, trabecular thickness, and bone volume/total volume ratio for Sr-β-CS compared to β-CS; histomorphometric analysis showed higher new bone area, blood vessel area, and faster in vivo degradation for Sr-β-CS compared to β-CS	[30]
**Akermanite** **(AK)**	Porous scaffolds	Rabbit femoral defects	8 and 16 weeks	Fluorescence labelling showed no significant difference in mineral apposition rate of new bone formation between AK and β-TCP scaffolds; histomorphometric analysis showed slightly higher new bone formation, and faster in vivo degradation of AK scaffolds compared to β-TCP	[98]
	Porous scaffolds	Ovariectomised rat calvarial defects	2, 4, 6 and 8 weeks	µ-CT analysis showed higher trabecular thickness and bone volume/total volume ratio in AK scaffolds compared to β-TCP; polychrome sequential fluorescent labelling showed enhanced new bone growth and mineral apposition in AK scaffolds compared to β-TCP; histomorphometric assay showed higher new bone area and blood vessel area in AK scaffolds compared to β-TCP	[100]
**Diopside (DP)**	Dense specimens	Rabbit jaw bone defects	12 weeks	Direct, gradient bonding between native bone and DP implant	[52]
	Dense spheres (1–1.5 mm diameter)	Rat femoral defects	2 and 4 weeks	Histological analysis showed new bone growth which formed tissue bridges with DP spheres, slightly higher bone regeneration score compared to β-TCP, and evidence of dynamic endochondral ossification; quantitative analysis on histology sections showed higher Col1 expression and similar OPN expression compared to β-TCP	[111]
**Hardystonite** **(HT)**	Porous scaffolds	Rat tibial defects	3 and 6 weeks	HT scaffolds showed new bone formation inside scaffold pores in both the external cortex and internal medullary cavity, in comparison to only external cortex for β-TCP control at both 3 and 6 weeks; limited in vivo resorption and limited ALP activity compared to β-TCP	[25]
**Sr-hardystonite** **(Sr-HT)**	Porous scaffolds	Rat tibial defects	3 and 6 weeks	Sr-HT scaffolds showed new bone formation inside scaffold pores in both the external cortex and internal medullary cavity, in comparison to only external cortex for β-TCP control at both 3 and 6 weeks; limited in vivo resorption but extensive ALP activity compared to hardystonite and β-TCP	[25]
**Baghdadite** **(Bag)**	Dense 1–1.5 mm diameter spheres	Rat femoral defects	2 and 4 weeks	Histological analysis showed new bone growth which formed tissue bridges with Bag spheres, significantly higher bone regeneration score compared to β-TCP, and evidence of dynamic endochondral ossification with increased amount of regularly arranged woven bone compared to diopside and β-TCP; significantly higher Col1 expression and OPN expression compared to diopside and β-TCP scaffolds	[111]
	Porous scaffolds	Rabbit radial segmental defects	12 weeks	Radiographic analysis showed enhanced defect bridging for Bag scaffolds compared to BCP scaffold; histological analysis showed enhanced bone ingrowth into pores of Bag scaffold compared to mostly peripheral bone growth for BCP scaffold; histomorphometric analysis showed increased new bone formation in Bag scaffolds (3.0 ± 3.1 mm^2^) compared to BCP (1.3 ± 1.0 mm^2^) at the scaffold midpoint; observed evidence of osteoclast-mediated resorption	[71]
	Porous scaffolds	Sheep tibial segmental defects	Up to 26 weeks	Radiographic analysis showed clinical union at the bone-scaffold interface in all samples after 26 weeks; biomechanical analysis showed that torsional strength of the implant and associated bone reached ~10% of contralateral intact tibia; histological analysis showed average 80% bridging of the defect length in all samples, as well as new bone growth inside the scaffold pores	[110]

CS: calcium silicate; β-TCP: β-tricalcium phosphate; BCP: biphasic calcium phosphate; Col1: collagen type I; OPN: osteopontin; ALP: alkaline phosphatase.
